# Genomic Selection for the Improvement of Antibody Response to Newcastle Disease and Avian Influenza Virus in Chickens

**DOI:** 10.1371/journal.pone.0112685

**Published:** 2014-11-17

**Authors:** Tianfei Liu, Hao Qu, Chenglong Luo, Xuewei Li, Dingming Shu, Mogens Sandø Lund, Guosheng Su

**Affiliations:** 1 College of Animal Science and Technology, Sichuan Agricultural University, Yaan, China; 2 Institute of Animal Science, Guangdong Academy of Agricultural Sciences, Guangzhou, China; 3 Center for Quantitative Genetics and Genomics, Department of Molecular Biology and Genetics, Aarhus University, Tjele, Denmark; 4 State Key Laboratory of Livestock and Poultry Breeding, Guangzhou, China; Icahn School of Medicine at Mount Sinai, United States of America

## Abstract

Newcastle disease (ND) and avian influenza (AI) are the most feared diseases in the poultry industry worldwide. They can cause flock mortality up to 100%, resulting in a catastrophic economic loss. This is the first study to investigate the feasibility of genomic selection for antibody response to Newcastle disease virus (Ab-NDV) and antibody response to Avian Influenza virus (Ab-AIV) in chickens. The data were collected from a crossbred population. Breeding values for Ab-NDV and Ab-AIV were estimated using a pedigree-based best linear unbiased prediction model (BLUP) and a genomic best linear unbiased prediction model (GBLUP). Single-trait and multiple-trait analyses were implemented. According to the analysis using the pedigree-based model, the heritability for Ab-NDV estimated from the single-trait and multiple-trait models was 0.478 and 0.487, respectively. The heritability for Ab-AIV estimated from the two models was 0.301 and 0.291, respectively. The estimated genetic correlation between the two traits was 0.438. A four-fold cross-validation was used to assess the accuracy of the estimated breeding values (EBV) in the two validation scenarios. In the family sample scenario each half-sib family is randomly allocated to one of four subsets and in the random sample scenario the individuals are randomly divided into four subsets. In the family sample scenario, compared with the pedigree-based model, the accuracy of the genomic prediction increased from 0.086 to 0.237 for Ab-NDV and from 0.080 to 0.347 for Ab-AIV. In the random sample scenario, the accuracy was improved from 0.389 to 0.427 for Ab-NDV and from 0.281 to 0.367 for Ab-AIV. The multiple-trait GBLUP model led to a slightly higher accuracy of genomic prediction for both traits. These results indicate that genomic selection for antibody response to ND and AI in chickens is promising.

## Introduction

Newcastle disease (ND) and avian influenza (AI) are regarded as two of the most important diseases of poultry worldwide and can lead to a flock mortality up to 100% [1]–[5]. An outbreak of ND or AI can cause a serious loss to the local economy. Fortunately, previous studies have shown that genetic selection might improve resistance to the diseases [6]–[8] and development of disease resistance through indirect selection primarily on immune response traits may be the best long-term strategy [9], indicating that it is possible to develop poultry lines with a high-level of biosecurity using immune response traits. Accurate genetic selection using conventional genetic evaluation methods has a high demand for accurate pedigrees and a large number of phenotypic records. However, such new lines usually only have a small population size, and it is difficult and costly to obtain the phenotypic records. Consequently, the accuracy of EBV of immune response traits is limited by using conventional methods. Genomic selection is a new genetic selection method, which directly incorporates markers throughout the genome to estimate breeding values [10]. With the development of high-throughput technology and the decrease of genotyping price, genomic selection has been widely used in animal breeding. Genomic selection especially benefits for the traits which are expensive to measure such as antibody response to Newcastle disease virus (Ab-NDV) and antibody response to Avian Influenza virus (Ab-AIV), since genomic selection can provide accurate prediction of breeding value for the individuals without their own records. Genomic best linear unbiased prediction models (GBLUP) or genomic Bayesian models are usually used in genomic selection. The main difference between GBLUP and Bayesian models is the assumption for distributions of SNP marker effects. The GBLUP models assume that effects of all markers are normally distributed with the same variance [11], and the Bayesian models generally assume that most markers have a null or very small effect, and a small number of markers have a larger or moderate effect [12]–[14]. Using simulated data, several previous studies showed that Bayesian models were superior to the GBLUP models [10], [15]. However, in some cattle and pig studies [16], [17], the GBLUP model performed as well as the Bayesian model for most traits. Meuwissen et al. [18] noted that because the real number of QTL is large, the assumption of every SNP being in LD with a QTL is about right. Several studies [19]–[23] have attempted to find some large effect QTL on Ab-NDV and Ab-AIV using association analysis. Although many significant markers have been detected, each of these markers has a small effect and none of these markers can explain more than 5% of the phenotypic variance. Immunological traits are complex traits, which are controlled by a large number of QTL with small effects. The GBLUP model may be a good approach to perform genomic selection for Ab-NDV and Ab-AIV because the traits may be affected by large number of QTL and the model has a low computational demand.

Previous genomic studies mainly focused on single-trait analysis; however, several traits of economic importance, which may be genetically related, were usually selected for animal breeding. Using the information from correlated traits, multiple-trait analysis could improve the accuracy of the estimated breeding value [24]–[26]. In the current study, genomic selection was carried out using single-trait and multiple-trait GBLUP models. To our knowledge, this is the first study to report genomic selection for Ab-NDV and Ab-AIV. The objectives of this study were to investigate the efficiency of genomic prediction for Ab-NDV and Ab-AIV based on genome-wide dense markers in a crossbred chicken population.

## Materials and Methods

### Ethics Statement

This study was approved by the Animal Care Committee of the Institute of Animal Science, Guangdong Academy of Agricultural Sciences (Guangzhou, People's Republic of China), the Approval No. is GAAS-IAS-2009-73. Blood samples of birds were collected from the brachial vein by standard venipuncture procedure. The chickens were treated humanely, and none of them were sacrificed for this study.

### Population and data

The chicken population in this study originated from a cross between two divergently selected lines, i.e., the “High Quality chicken Line A” (HQLA) and the Huiyang Beard chicken (HB), as described in Sheng et al [27]. A 3-generation pedigree included 20 individuals of F0 generation, 51 individuals of F1 generation and 511 birds of F2 generation. All individuals were genotyped and individuals of the F2 generation had phenotypic records.

Chickens were vaccinated with commercial vaccines in accordance with the instructions. At 25 days of age, the chickens were vaccinated against Newcastle disease with a commercially available LaSota strain (Intervet International B.V., Boxmeer, Netherlands) using the eye drop technique. After 25 days, a second vaccination against Newcastle disease was implemented. At 40 days of age, the chickens were vaccinated with a commercially Avian Influenza Inactivated H9 strain Vaccine using the eye drop technique. At 91 days of age, blood samples were collected. NDV and AIV antibody levels in the blood samples were measured using indirect ELISA and were expressed as the S/P value of the corresponding dilutions, according to the instructions for the commercial ELISA kit (BioCheck, Inc., Foster City, CA, USA). Table 1 shows the means and standard deviations of Ab-NDV and Ab-AIV for the 511 F2 birds. As shown in Table 1, the two traits had a markedly skewed distribution. To meet the model assumption of normally distributed residuals, a Box-Cox transformation [28] was applied,

**Table 1:** Mean and standard deviation of antibody response to Newcastle disease and Avian Influenza virus.

Trait^1^	N	Mean	SD	Min value	Max value
Ab-NDV	511	3.63	1.57	0.67	9.10
Ab-AIV	511	1.31	1.18	0.09	11.02

1 Ab-NDV  =  antibody response to Newcastle disease virus; Ab-AIV  =  antibody response to Avian Influenza virus.



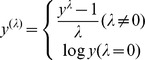
.

A set of λ was investigated. Finally, parameter λ was set as 0.05 and 0.5 for Ab-NDV and Ab-AIV, respectively. After transformation, the two traits met normal distribution and the analysis was based on the transformed data.

The birds were genotyped using the Illumina Chicken 60K SNP Beadchip [29], at DNA LandMarks Inc., Saint-Jean-sur-Richelieu, Canada. The SNP data were edited using criteria in the following order: call rate more than 95%; Gentrain scores more than 0.4; minor allele frequency more than 0.01. After editing, 46,672 SNP markers were retained.

### Statistical Models

In this study, breeding values were estimated using two models, i.e., a pedigree-based best linear unbiased prediction model (BLUP) and a genomic best linear unbiased prediction model (GBLUP). Both single-trait and multiple-trait analysis were implemented. Hereafter single-trait analysis is denoted as BLUP_ST_ or GBLUP_ST_, and multiple-trait analysis as BLUP_MT_ or GBLUP_MT_, respectively.

#### BLUP Model

The pedigree-based BLUP model [30] is as follows: 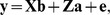 where **y** is the vector of the Box-Cox translation data of Ab-NDV and Ab-AIV, **b** is the vector of the fixed effect including sex and hatch,**a** is a vector of additive genetic effects, **e** is the vector of random residuals, **X** and **Z** are incidence matrices. It is assumed that 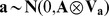,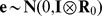, where **A** is the additive genetic relationship matrix built using pedigree information in which unknown parents of birds in the two parental lines are treated as two different genetic groups, **V_a_** is the covariance matrix of additive genetic effects, and **R_0_** is residual covariance matrix. **V_a_** and **R_0_** is a scalar in single-trait analysis and has a dimension of two in multiple-trait analysis.

#### GBLUP Model

The GBLUP model based on genomic information is: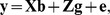 where **y, b, X** and **e** are the same as those in the BLUP model, **g** is a vector of genomic breeding values to be estimated, **Z** is the incidence matrices of **g**. It is assumed that 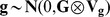, where **G** the additive genetic relationship matrix based on SNP markers[31], **V_g_** is the covariance matrix of genomic breeding values.

The variance and covariance components were estimated using Average Information Restricted Maximum Likelihood (AIREML) [32], and the analysis of BLUP model and GBLUP model was carried out using DMU package [33].

### Cross-validation

A four-fold cross validation was used in this study. Two scenarios were considered with regard to training and validation sets. The first cross validation scenario is random family sampling (CVF), in which all of the data for 8 half-sib families were randomly divided into four subsets, i.e., each subset has 2 half-sib families. The second cross validation scenario is random individual sampling (CVR), in which all of the data (511 birds) were randomly split into four subsets. In each fold of validation, one data set was used as the test data set and the other three data sets as training datasets. In the family sample scenario, the test birds did not have any sibs as training birds, and thus had a distant relationship to the training birds. Conversely, in the random sample scenario, the test birds had many sibs as training birds, and thus had a close relationship with the training birds. To account for population structure and sampling variation, splitting was repeated 10 times in CVF and 50 times in CVR. The numbers of birds in the test and training data sets for each fold of validation are shown in Table 2.

**Table 2:** Number of birds in the training and test data sets of the 4-fold cross-validation in the scenarios of family sample and random sample.

Scenario^1^	Training data	test data	Scenario	Training data	test data
CVF_fold1	381	130	CVR_fold1	383	128
CVF_fold2	383	128	CVR_fold2	383	128
CVF_fold3	385	126	CVR_fold3	383	128
CVF_fold4	384	127	CVR_fold4	384	127

1 CVF  =  the cross validation scenario by random family sampling (an example from 10 repeats); CVR  =  the cross validation scenario by random individual sampling.

In this study the accuracy of prediction was defined as the correlation between prediction and corrected phenotypic value (y_c_), where y_c_ was calculated as the phenotypic value corrected for fixed sex and batch effects. A paired t test was implemented to test the differences among the correlations obtained from these prediction models. The paired t test treated a fold of validation as a subject and took a pair of correlation coefficients for the fold from two models as a matched pair of observations.

## Results

### Estimates of variance components and heritability

As shown in Table 3, Ab-NDV had a moderately high heritability. Using the pedigree information, the heritability was 0.478 and 0.481 as estimated from BLUP_ST_ and BLUP_MT_, respectively. Ab-AIV had a moderate heritability of 0.301 and 0.291 as estimated from BLUP_ST_ and BLUP_MT_, respectively. The phenotypic correlation between Ab-NDV and Ab-AIV was 0.419, and the additive genetic correlation was 0.438. Heritabilities estimated from marker-based models were lower than those from pedigree-based models for Ab-NDV, and higher for Ab-AIV. However, the differences were not statistically significant.

**Table 3:** Estimates of additive genetic variance, residual variance and heritability using a single-trait and a multiple-trait linear mixed model based on the full data.

Trait^1^	Method	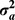	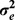	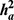
Ab-NDV	BLUP_ST_	0.306	0.335	0.478±0.142**
	BLUP_MT_	0.314	0.330	0.487±0.144**
	GBLUP_ST_	0.206	0.367	0.360±0.075**
	GBLUP_MT_	0.210	0.364	0.366±0.073**
Ab-AIV	BLUP_ST_	0.142	0.329	0.301±0.123*
	BLUP_MT_	0.136	0.332	0.291±0.120*
	GBLUP_ST_	0.158	0.291	0.351±0.077**
	GBLUP_MT_	0.155	0.292	0.347±0.077**

1 Ab-NDV  =  antibody response to Newcastle disease virus; Ab-AIV  =  antibody response to Avian Influenza virus.

^*^ Significantly different from 0 at P<0.05.

^**^ Significantly different from 0 at P<0.01.

### Accuracy of predictions using different models

Table 4 shows the accuracy of predictions using different models. In CVF, the BLUP models (BLUP_ST_ and BLUP_MT_) had a low predictive ability. As expected, the genomic models (GBLUP_ST_ and GBLUP_MT_) performed better than the conventional BLUP models. The accuracies between the conventional model and genomic model were significantly different. In CVR, the gain in predictive ability by using marker information was not as large as in CVF, but the difference between the conventional model and the genomic model was still significant.

**Table 4:** Correlations between corrected phenotypic values and genomic predictions for the birds in the test sets of the cross-validation.

Trait^1^	Scenario^2^	BLUP_ST_	BLUP_MT_	GBLUP_ST_	GBLUP_MT_	Scenario	BLUP_ST_	BLUP_MT_	GBLUP_ST_	GBLUP_MT_
Ab-NDV	CVF_mean	0.086^a^	0.087^a^	0.223^b^	0.237^b^	CVR_mean	0.391^a^	0.389^a^	0.424^b^	0.427^b^
	CVF_fold1	0.093	0.099	0.249	0.270	CVR_fold1	0.402	0.399	0.433	0.439
	CVF_fold2	0.064	0.072	0.182	0.206	CVR_fold2	0.402	0.401	0.435	0.440
	CVF_fold3	0.138	0.128	0.229	0.239	CVR_fold3	0.379	0.379	0.410	0.412
	CVF_fold4	0.050	0.049	0.233	0.234	CVR_fold4	0.379	0.377	0.417	0.418
Ab-AIV	CVF_mean	0.094^a^	0.080^a^	0.332^b^	0.347^c^	CVR_mean	0.284^b^	0.281^a^	0.364^c^	0.367^d^
	CVF_fold1	0.071	0.066	0.360	0.383	CVR_fold1	0.298	0.295	0.375	0.378
	CVF_fold2	0.073	0.034	0.306	0.313	CVR_fold2	0.292	0.289	0.367	0.368
	CVF_fold3	0.061	0.064	0.345	0.364	CVR_fold3	0.277	0.275	0.359	0.364
	CVF_fold4	0.170	0.155	0.316	0.328	CVR_fold4	0.268	0.266	0.354	0.359

1 Ab-NDV  =  antibody response to Newcastle disease virus; Ab-AIV  =  antibody response to Avian Influenza virus.

2 CVF  =  the cross validation scenario by random family sampling; CVR  =  the cross validation scenario by random individual sampling.

a–d within a row, estimates without a common superscript differ significantly (P<0.05), according to the paired t test.

In single-trait analysis, compared with conventional EBV, the accuracies of the genomic predictions from GBLUP_ST_ for Ab-NDV and Ab-AIV increased by 0.137 and 0.238 in CVF, and 0.033 and 0.080 in CVR, respectively. In multiple-trait analysis, in contrast to conventional EBV, the accuracies of the genomic predictions from GBLUP_MT_ for Ab-NDV and Ab-AIV increased by 0.150 and 0.267 in CVF, and 0.038 and 0.086 in CVR, respectively. For both traits, the multiple-trait model (GBLUP_MT_) led to an increase in accuracy of genomic prediction.

## Discussion

### Heritability and genetic correlation

Cross-breeding is widely applied in beef, pigs and chickens [34]–[38], and new pure lines are usually developed by cross-breeding [39]. However, the heritability of a trait in a crossbred line is not necessarily in line with the heritability in a purebred line. In laying chickens, Wei et al. [38] analyzed data from two White Leghorn purebred lines and their crossbred line, and compared the heritability between purebred and crossbred lines. Among the seven traits, purebreds showed higher heritability. For egg weight at 30 to 35 weeks of age, the heritability was 0.63 in purebreds, and 0.27 in crossbreds. For egg specific gravity at 30 to 35 weeks of age, the heritability was 0.83 in purebreds, and 0.13 in crossbreds. Gavora et al. [40] reported the heritability of resistance to Marek's disease in White Leghorn strains was 0.85 in a pure-strain and 0.45 in a cross-strain. These results indicate that in general the heritability in purebreds could be similar to or higher than that in crossbreds. In the current study, the population was crossbred, so it is expected that the heritability in purebreds could be similar or higher. In the present study, the heritability was 0.48 for Ab-NDV. However, Sacco et al. [41] reported the heritability of Ab-NDV was only 0.30 in a purebred turkeys. This is likely because genetic variation of F2 birds usually is larger than in the F1 generation. Additionally, different populations may have different heritability for the Ab-NDV. In this study, the results showed that Ab-NDV and Ab-AIV have moderate heritability, which indicates that the improvement of resistance to NDV and AIV by selection might be effective.

In addition, the present study showed a favorable moderate genetic correlation between Ab-NDV and Ab-AIV, which indicates that selection for one trait will led to a correlated favorable response for the other trait.

### Accuracy of genomic selection for antibody response

Genomic prediction based on genome-wide dense markers captures not only the information about linkage disequilibrium between markers and QTL but also the additive genetic relationship between individuals [11]. Although conventional selection has been shown to effectively improve resistance to Marek's disease [42] and improve leg health in broilers [43], it is expected that genomic prediction is more accurate than conventional pedigree-based prediction. Currently genotyping is still costly. Fortunately, the cost of genotyping move swiftly downward and it is expected that genomic selection will be widely applied in poultry breeding schemes. Comparisons between genomic prediction and conventional prediction have been performed in various livestock species, such as cattle [44], [45] and chickens [46], [47]. These studies reported similar results, i.e., genomic prediction methods were superior to the conventional prediction method. However, in a study on resistance to the Salmonella carrier state in laying hens by Legarra et al. [48], the accuracy of genomic prediction based on 1536 SNP markers was not higher than conventional BLUP model. The authors argued that denser markers were needed to improve the accuracy of genomic prediction. In the current study, markers on the 60K SNP Beadchip were used in the models to predict breeding value for Ab-NDV and Ab-AIV. The results showed that genomic prediction is superior to the conventional method. Genomic prediction for Ab-NDV and Ab-AIV based on genome-wide dense markers is more efficient in a crossbreed chicken population.

The Bayesian model is another type of model that was widely implemented in genomic selection. Several previous studies have reported differences in the accuracy of genomic prediction between GBLUP and Bayesian models. Su et al. [49] compared a GBLUP model with a Bayesian mixture model with 2 distributions in Nordic Holsteins, and reported that the Bayesian mixture model performed better than the GBLUP model. However, Ostersen et al. [17] studied the reliabilities of the genomic prediction for daily gain and feed conversion ratio using a GBLUP model and a Bayesian mixture model with 2 distributions in the Danish Duroc pig population, and reported that the Bayesian model did not perform better than the GBLUP model. The authors argued that a GBLUP model would perform well for a population in which there are strong relationships between animals. A Bayesian mixture model with 4 distributions was implemented the in present study (results not shown). The Bayesian model led to a slightly higher reliability than GBLUP in CVF (distant relationship), but not in CVR (close relationship). Nonetheless, the multiple-trait model constantly performed better than the Bayesian single trait model. This implies that the number of QTL of Ab-NDV and Ab-AIV are large, and the two traits are controlled by many small effect genes. A genome-wide association study using the Bayesian mixture model detected 32 and 45 markers which were statistically significantly associated with Ab-NDV and Ab-AIV, respectively, but each of these markers has a small effect and none explained more than 5% of the phenotypic variance (results not shown).

In this study, the data were split into training and test data sets in two different ways, and cross-validation was carried out based on these data sets to evaluate the accuracy of genomic predictions. The accuracies in CVR were higher than those in CVF. This is because, in contrast with CVF, the test birds had sibs in the training data in CVR. Therefore, the genetic ties between test and training birds are much stronger in CVR, which confirms that the accuracies were higher with more close genetic relationship between test and training animals, as reported by previous studies [44], [45], [50], [51]. In practical breeding schemes, some closely related individuals were available between the reference and candidate population, then the accuracies of GEBV could be fall between the accuracies of CVF and CVR.

### Comparison of single-trait and multiple-trait genomic prediction models

Comparisons between single-trait and multiple-trait models for genomic prediction have been reported in previous studies. Christensen et al. [52] compared accuracies of predicted breeding values for the daily gain and feed conversion ratio in Danish Duroc pigs using single-step models, which use information from genotyped and non-genotyped animals simultaneously. They reported that the multiple-trait analysis gave higher accuracy only for the feed conversion rate of non-genotyped animals. In a simulation study, Guo et al. [24] observed that a multiple-trait model improved genomic prediction for a trait with few records if a correlated trait with a large number of records existed. In the current study, the multiple-trait analysis (GBLUP_MT_) gave better genomic prediction than the single-trait analysis (GBLUP_ST_), which was more pronounced in CVF. This suggests that the extra information from the correlated trait would be relatively more important when the information from relatives is weaker.

Genetic correlations among traits impacts the accuracy of the multiple-trait model. Using simulation data, Calus et al. [53] studied the accuracies of multiple-trait genomic prediction. They reported that when using a multiple-trait model, the accuracy of genomic prediction increased as the genetic correlation between traits increased. When the genetic correlations between a low heritability trait and a high heritability trait were 0.25, 0.54 and 0.75, the accuracies of the low heritability trait increased by 0.02, 0.07 and 0.13, respectively. Similar results were reported by Jia et al. [25]. The heritability of the trait is another factor affecting the performance of the multiple-trait model. The trait with low heritability benefits more from a multiple-trait model. Guo et al. [24] reported that for the low heritability trait (h^2^ = 0.05) the reliability of GEBV using multiple-trait model increased up to 0.07 compared with the reliability using single-trait model. In contrast, for the higher heritability (h^2^ = 0.30) the reliability of GEBV did not improve. Jia et al. [25] argued that low heritability traits can borrow information from correlated high heritability traits and achieve higher accuracy, which is in line with Calus et al. [53]. In the present study, the genetic correlation between Ab-NDV and Ab-AIV was moderate, which not surprisingly resulted in higher accuracies of GEBV by using a multiple-trait model. Furthermore, the gain for the two immune traits using a multiple-trait model was similar. This was expected as the difference of heritability for the two traits was small, and all birds have records for both traits.

## Conclusion

This is the first study on genomic prediction for Ab-NDV and Ab-AIV. It was found that Ab-NDV and Ab-AIV were moderately heritable. Genomic prediction can greatly improve the accuracy of estimated breeding values. The genomic prediction using the multiple-trait model was more accurate than prediction using the single-trait model. The results indicate that genomic selection for Ab-NDV and Ab-AIV is promising.
